# Narcissism as a Moderator of Satisfaction with Body Image in Young Women with Extreme Underweight and Obesity

**DOI:** 10.1371/journal.pone.0126724

**Published:** 2015-05-11

**Authors:** Małgorzata Lipowska, Mariusz Lipowski

**Affiliations:** 1 Institute of Psychology, University of Gdansk, Gdansk, Poland; 2 Department of Health Psychology, Gdansk University of Physical Education and Sport, Gdansk, Poland; University of Udine, ITALY

## Abstract

**Objective:**

Body weight and age constitute main determinants of body image in women. We analyzed the role of narcissism as a moderator of body image in young women representing various extremes of body weight.

**Methods:**

The study included 325 women between 18 and 35 years, qualified into three BMI categories: obese women (BMI > 30.0, *n* = 72), severely underweight women who did not satisfy the remaining criteria of anorexia (BMI < 17.5, *n* = 85), and women with normal body weight (21.7 < “ideal BMI” > 22.7, *n* = 168). Satisfaction with body image was determined with Multidimensional Body-Self Relations Questionnaire and Body Esteem Scale, while narcissism was measured with Narcissistic Personality Inventory.

**Principal Findings:**

We revealed that narcissism has significant impact on the body image of women who are extremely underweight or obese. *Vanity* and *Leadership* were narcissism dimensions which played significant role in slim women, as compared to *Vanity* and *Self-Sufficiency* in obese women.

**Conclusion:**

The role of narcissism as a modulator of self-satisfaction with one’s body varies depending on BMI level: extremely underweight women and obese individuals constitute groups in which narcissism has the strongest impact on the self-satisfaction with body.

## Introduction

Perception of one’s body as attractive is associated not only with its objective parameters (height, weight and proportions) [1–5], but also with cultural canons [6–10] and fashion [11–13]. Furthermore, one’s self-satisfaction with body image is also modulated by a subjective attitude to bodily sphere, which in turn is determined by individual characteristics, such as personality profile [14,15], general self-esteem [16], psychological gender [17,18], optimism [19], locus of control [20,21] and narcissism [22–24].

Research suggests [25] that self-attitude to one’s body, which is based on cognitive, behavioral, and emotional components and the subjective determination of the size of various body parts, represents two relatively independent elements of body image. Age and gender are the two factors which most significantly diversify the way the body is felt and perceived, as well as the role of this process. According to the general developmental trend, the level of body-esteem changes throughout the lifetime. After the period of reaching puberty and then adulthood, when it falls quite deeply due to external opinions [26–28], it gradually increases during middle adulthood [29–32], and plummets at the threshold of the old age as a result of confrontation with the evident signs of aging and the general, often negative, opinions about effects of aging [33]. During the period of adolescence and adulthood, beauty is associated mostly with interpersonal success and constitutes an important determinant of social prestige and appreciation; as seems to emanate from each advertisement [34].

Only few psychological processes are more gendered than body image. Men and women differ in both content and degree of body dissatisfaction and body change behaviors [34–37]. Currently gender differences are clear and feasible: while females focus on body weight and body shape, males center around the muscular apparatus [26,38,39]. However the most important difference refers to the subjective significance of physical attractiveness weighted by women and men [37,40,41].

Negative self-attitude of women to their bodies is usually associated with low self-esteem which is observed irrespective of age [30,42,43]. Women are dissatisfied with certain aspects of their image and can even consider their body weight as a determinant of their image [37,44]. Current fashion of slenderness has been reflected by the popularity of various slimming diets; they are most commonly applied to address the appearance of any “defects” of the body and are used by individuals with feelings of being obese, even despite the lack of objective reasons [45–47]. Nevertheless, concluding that body mass is being overestimated and we represent a community of slim individuals, who just assess themselves inadequately, would be a misinterpretation. Unfortunately, there is a growing evidence of obesity being a civilization-related problem of the 21^st^ century. Interestingly, the promotion of slim or even cachectic silhouette in the media is associated with higher incidence of eating disorders; although, it is accompanied by a rapid increase in the number of obese individuals, and the worldwide obesity rate has more than doubled since 1980 [48]. In this matter, we face the so-called “scissors phenomenon” – growing number of underweight women and girls on one hand, and increasing risk of overweight on the other. Aside from individuals suffering from overweight or obesity, who should lose weight and have a history of multiple unsuccessful attempts at accomplishing this goal, there is a growing number of subjects trying to lose weight (frequently with success) despite evident underweight. While only 3% of Poles are severely underweight, as many as 30% of Polish women and 45% of men are overweight (BMI 25.00–29.99) and 15% and 16% of them are obese (BMI > 30) [49]. In United States, 33.0% of women and 35.5% of men are obese [50], while 1.7% are underweight (BMI < 18.5) [51]. Body dissatisfaction is positively correlated with body weight, both in a group of healthy women and in those suffering from eating disorders [52]. The higher is the level of dissatisfaction, the more frequently the body is perceived as larger than it really is; individuals trying to lose weight overestimate their size and this conviction of being fat rather than overweight is the source of dissatisfaction [21,39].

Aside from the parameters being directly associated with one’s bodily sphere, self-satisfaction with one’s body image is also determined by a number of personality predispositions, as well as by the relationship between personality traits and sensitivity to suggestions from others, including mass media [53,54]. By concentrating on their image, many individuals lose touch with reality and apply various beauty treatments or surgical corrections despite receiving feedback from close relations indicating that they worsen rather than improve their appearance [55]. Additionally, not infrequently, despite the lack of objective reasons, such individuals insist on being complemented on their appearance. Such insistence on appreciation and admiration from others despite lacking merits is one of the features of narcissism, understood as a personality dimension [56]. “Narcissism” has unambiguous negative connotation in everyday use and refers to those who have fallen in love with himself/herself and love only himself/herself. However, being proud and satisfied with ourselves and our image is not a sign of any pathology, but rather a form of self-acceptance required for normal development; we are unable to function and develop normally without it [57]. If an individual manifests his/her self-dependence and is convinced of his/her comprehensive and undisputed knowledge to a degree that he/she is unable to experience fear, humility, joy, grief, jealousy, uncertainty, or helplessness – which has significant negative impact on his/her interpersonal relationships – the pathological narcissism can be suspected [58]. Where is the borderline between the norm and the disorder? Although the normal and pathological narcissism are sometimes considered as the extremes of the same continuum (e.g. [59]), a growing body of empirical data and theoretical meta-analyses suggest that they represent two independent entities [60]. Both types of narcissism are underlied by a need for admiration and recognition, but differ in terms of mature regulatory mechanisms level; narcissistic behaviors can have many forms and be either adaptive or maladaptive [61]. In the case of pathological narcissism relations with other people are predominated by the feeling of jealousy, which is associated with a tendency for devaluing others, for example by judging their skills, lack of interest, and appropriating achievements. Moreover, narcissistic individuals are unable to engage in interpersonal relationships, mostly due to the fear of being dependent on other people, or lack of empathy [62,63]. Maintenance of relationships with other people is based on the manifestation of self-superiority and self-dominance. The narcissistic individual needs people to strengthen his/her self-love, and therefore strives to be surrounded by people who will admire him/her uncritically and strengthen his/her conviction of being someone unusual. The Narcissistic Personality Disorder, the diagnostic criteria of which are included in DSM-5 [64], represents evident example of pathological narcissism. Although the association between normal narcissism and positive self-esteem, well-being [65], high level of aspirations, and leadership skills [66] is emphasized, also a tendency of narcissistic individuals to positive self-assessment of their behaviors despite the presence of contradictory facts is highlighted [67].

## Objectives

Since body weight is a principal determinant of female body image, one should compare attitudes to the latter in women with different BMIs corresponding to obesity, severe underweight and ideal body weight.

Furthermore, taking into account that preoccupation with self-appearance can be regarded as one of the signs of narcissism, it would be interesting to analyze the relationship between narcissism, considered as a personality trait, and self-satisfaction with one’s appearance. In view of the theoretical background of narcissism construct, it can be assumed that there is a positive relationship between the level of this trait and positive self-evaluation of one’s body image. Furthermore, it is of interest if narcissism in general, or any of its component traits, can affect the self-perception of one’s attractiveness. Finally, we would like to determine if the impact of narcissistic traits on the self-esteem can be modulated by the degree of one’s body weight.

Researchers analyzing the role of personality predispositions as determinants of self-satisfaction with body image, usually adjust the results for body weight of their subjects. The results of the hereby presented study were not only adjusted for the objective parameter (BMI); we went one step further and enrolled participants representing different BMI categories in order to accurately determine the role of narcissism as a determinant of body image among women with various types of silhouette.

## Methods

The protocol of this study was approved by the Ethics Board for Research Projects at the Institute of Psychology, University of Gdansk (decision no. 4/2010). Prior to the study written informed consent was obtained from all participating women.

## Participants and Procedure

Verification of the relationship between narcissism and body image requires controlling for variables that can significantly modulate the level of life satisfaction. Obviously, these variables include age [43,44] and gender [37]. Therefore, we decided to include only young women between 18 and 35 years (*M* = 24.1, *SD* = 5.5) in our study. Defining such limit of age prevented the confounding effect of this factor. On the basis of predefined inclusion criteria (age and gender) we selected the study group among 4 560 individuals taking part in the population-based study entitled *Psychological Determinants of Body Image*, including randomly selected Three-City (Gdansk-Sopot-Gdynia; Poland) inhabitants.

The next selection criterion was body weight, mentioned previously as a determinant of self-assessed beauty [25,39]. Our study included 325 young women representing various extremes of body weight. Anthropometric measurements were taken by individuals involved in the project, i.e. its authors and auxiliary personnel. *Body Mass Index* (BMI), i.e. the ratio of body weight to body height calculated on the basis of objective anthropometric measurements, was the qualification criterion to the analyzed groups. Although this measure is very popular, it should be remembered that it does not reflect the type of body structure, namely the muscle mass to fat mass ratio. Therefore, women who practiced sports on a professional basis were excluded from our study as their results could be overestimated (the weight of muscle tissue is higher than that of adipose tissue). None of the participants have been practicing any sport on a competitive basis, either at the time of the study or in the past. The participants were qualified into three BMI categories: obese women (*n* = 72, BMI > 30.0, *M* = 33.13, *SD* = 3.39), severely underweight women (*n* = 85, BMI < 17.5, *M* = 16.64, *SD* = 1.05), and women with BMI between 21.7 and 22.7 (*n* = 168, *M* = 21.52, *SD* = 0.59), i.e. in the exact center of range corresponding to normal body weight (referred to as “ideal BMI” group). We have selected these three groups, representing the extremes and exact center of the BMI continuum, purposefully. The aim was not to confirm the role BMI plays in determining satisfaction with one's appearance but rather to identify potential differences in the influence of narcissism on body image perception resulting from the degree of obesity/thinness in extreme cases. Importantly, women with BMI < 17.5 did not satisfy other diagnostic criteria of anorexia included in DSM-IV-TR [68]. Participants qualified to this group were additionally referred to clinical consultation which ultimately excluded anorexia on the basis of comprehensive evaluation. As a result, all women with anorexia were excluded from the study.

## Measures

Attitude of our participants towards their bodies was determined with two tests: Multidimensional Body-Self Relations Questionnaire [69] and Body Esteem Scale [70], while the level of their narcissistic traits was examined with Narcissistic Personality Inventory [71], the most widely used measure of narcissism in normative samples [61]. Using two different tests for self-attractiveness enabled us to determine the overall attractiveness score (in MBSRQ) and its particular components (BES).

### The Multidimensional Body-Self Relations Questionnaire

The Multidimensional Body-Self Relations Questionnaire [69] comprises 69 questions grouped into nine subscales. Only the subscales *Appearance Evaluation* and *Appearance Orientation* were used in our study, since the remaining subscales refer to *Fitness* and *Health*. Evaluation subscale verifies the self-attitude (positive/negative) to body image, whereas the Orientation subscale determines if the subject shows any activities to improve his/her body image. The final result of each subscale can be scored as very low, low, moderate, high, and very high. Polish adaptation of MBSRQ is based on the translation of the original version. The original survey was translated into Polish by two independent translators: an English philologist and a psychologist. Subsequently, the optimal version of the translation was developed as a result of consensus between these two specialists, and subjected to back-translation into English by a native speaker who was blinded to the original instrument. The consistency of the back translation and the original version was assessed by a bilingual translator. Due to specificity of Polish language, two separate versions of the questionnaire (for women and men) were developed. However, these versions differ solely in terms of gender-specific grammatical structures.

### The Body Esteem Scale

The Body Esteem Scale [70] examines the self-perceived body esteem. The scale comprises 35 items grouped into three, gender specific subscales. The subscales for women include *Sexual Attractiveness*, *Weight Concern*, and *Physical Condition*, whereas the body esteem of men is examined with regards to *Physical Attractiveness*, *Upper Body Strength*, and *Physical Condition*. Each BES statements can be scored using a five-item Likert-type scale, where 1 corresponds to *have strong negative feelings*, 5 to *have strong positive feelings*, and 3 represents a neutral midpoint. In women, the *Sexual Attractiveness* subscale refers to the perception of body components whose image cannot be modified by physical exercise (e.g. shape of lips, breasts). The attitude towards these body parts is associated with the emphasis of female sexuality, and their image can be modified solely by cosmetic procedures (e.g. makeup). In contrast, *Weight Concern* subscale refers to completely different components of the appearance; namely, body parts whose image can be improved by physical exercise or diet. Finally, the third subscale, *Physical Condition*, pertains to such parameters as stamina, strength, and agility. Polish adaptation of BES followed the same procedure as described above in the case of MBSRQ. Additionally, a reference range of the scale’s values was redefined upon obtaining consent from the author of the original version [72].

### Narcissistic Personality Inventory

**Fig 1:**
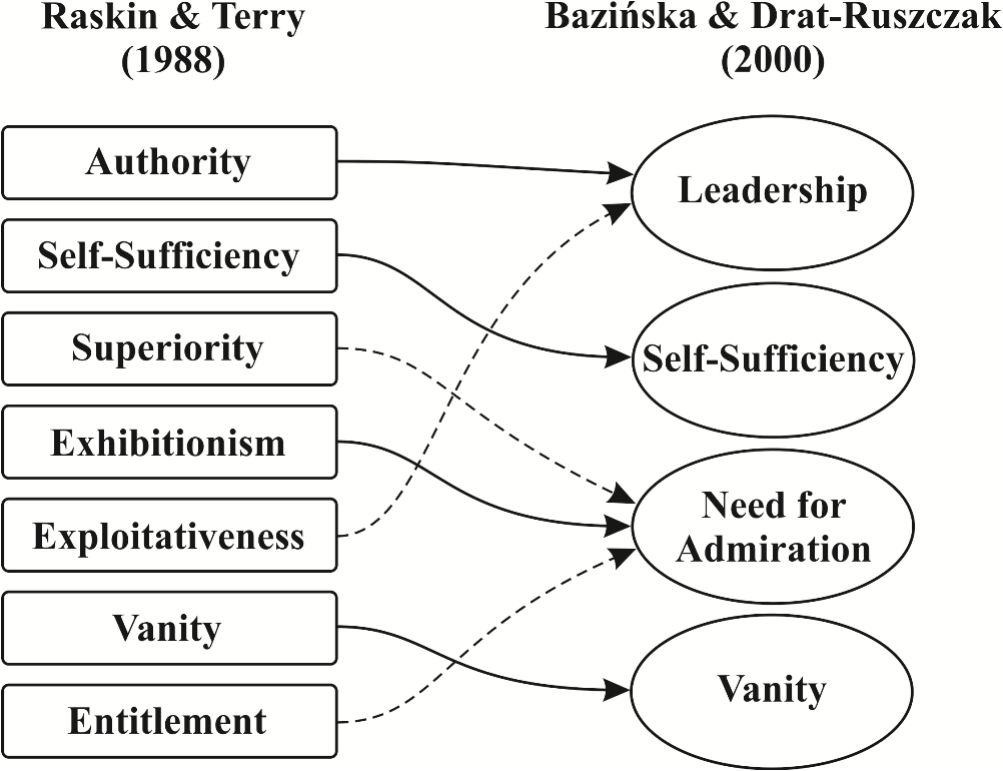
Items included in the original version and Polish adaptation of Narcissistic Personality Inventory (dashed arrow means modification of a scale).

Narcissistic Personality Inventory [71], in the form of Polish adaptation by Bazińska and Drat-Ruszczak [73] was used to determine the intensity of narcissism considered as a personality variable. The authors of the Polish adaptation developed it on the basis of questionnaires proposed by Emmons [74] and Raskin & Terry [75]. The initial version of the questionnaire developed by Bazińska & Drat-Ruszczak [73] comprised 44 items, all of them included in the instruments of Emmons [74] and Raskin & Terry [75]. The Polish adaptation was validated in three independent groups of subjects (*n*_1_ = 510, *n*_2_ = 504, *n*_3_ = 818). The final version of the instrument comprises four scales: *Need for Admiration*, *Leadership*, *Vanity*, and *Self-Sufficiency*, identified as most accurate during the adaptation procedure of the original seven-item scale to Polish conditions (Fig 1). Statistical analyses revealed satisfactory internal consistency of the four-item instrument (*Cronbach-alpha* for *Need for Admiration*, *Leadership*, *Vanity*, and *Self-Sufficiency* equal to 0.86, 0.86, 0.76, and 0.70, respectively), and factorial structure of the test provides its high stability. Two of the adapted scales, *Self-Sufficiency* and *Vanity*, are nearly identical to their originals, and the remaining two, *Leadership* and *Need for Admiration*, resemble their original counterparts (i.e. *Authority* and *Exhibitionism*) albeit were added the “manipulation- /entitlement-related” positions left after elimination of items with insufficient psychometric values. The manipulation-related statements, originally included in *Exploitative* scale, were added to *Leadership* scale. The entitlement-related statements and the statements emphasizing one’s self-superiority (included in *Superiority* and *Entitlement* scales of the original instrument) were added to *Need for Admiration* scale. In the adaptation of Bazińska & Drat-Ruszczak [73], *Leadership* corresponds to one’s conviction of having influence on the others, and *Need for Admiration* is associated with the assumption that admiration and appreciation from the others are not necessarily proportional or dependent on one’s achievements.

The *Need for Admiration* scale reflects the need of being noted, admired, complemented and famous, and includes an element of entitlement. This scale co-exists with the expectation of life satisfaction, indirect aggression (in women), suspicion (in men), self-consciousness in interpersonal relationships, pursuit of career, and the use of self-complication strategy. The *Leadership* scale determines the belief in the ability to influence others and self-assessed leadership capabilities. It co-exists with stable, high self-assessment, extraversion, indirect and verbal aggression (in women), purposiveness and self-consciousness in interpersonal relationships, and pursuit of career. The *Vanity* scale analyzes self-preoccupation with body image, self-admiration of physical image, sense of uniqueness and aestheticism. This scale is associated with high self-assessment, expectation of life satisfaction, and, in women, with a lack of guilt and social naivety. The *Self-Sufficiency* scale examines the self-conviction of competence, success, and independence. It co-exists with pursuit of career and independence from external, social standards of regulation [73].

## Results

We begin the presentation of our findings by describing the differences in body image assessment documented by means of *ANOVA* in women with various BMI. The results of *Levene Test* confirmed the homogeneity of variance of all studied variables (0.17 < *F* < 2.73,. 067 < *p* <. 843). The results obtained with the Multidimensional Body-Self Relations Questionnaire suggest that obese women score their body image the poorest (*low*) in *Appearance Evaluation Scale* (Table 1), while the scores of remaining participants appear to be moderate [62]. More detailed data was obtained by analyzing the results of the *Body Esteem Scale*, revealing that our women varied in assessing their image using various subscales of this instrument. It is of note that women with dissimilar BMI did not differ with regards to self-perceived sexual attractiveness; this parameter was scored moderately in all studied groups. It was not surprising that participants with disparate BMI scored differently in the second subscale of BES – *Weight Concern*. Our assumptions were confirmed by the results of *post-hoc* analysis (*Tukey Test* for unequal sample sizes): the slimmest women scored the highest and obese women the lowest (*p* <. 001 for both the comparisons). In turn, the analysis of intergroup differences revealed that women with BMI < 17.5 did not differ from those with moderate BMI with regards to *Weight Concern*. Women with “ideal” BMI scored the highest with regards to *Physical Condition*. In contrast, obese women scored significantly lower in terms of *Physical Condition*, compared to both the slimmest participants (*p* =. 027) and those with “ideal” BMI (*p* =. 007). This clearly suggests that the result of the analysis of variance was mostly determined by the “outlaying” of obese women in *Weight Concern* and *Physical Condition* subscales. We did not find any significant differences between women with diverse BMI with regards to the second analyzed scale, i.e. Multidimensional Body-Self Relations Questionnaire – *Appearance Orientation*. Of note, all our participants scored *moderate* in this scale.

**Table 1:** Differences in the self-perception of attractiveness in women with different BMI.

Scale	Scale min-max	BMI < 17.5: *M* (*SD*)	21.7 < BMI < 22.7: *M* (*SD*)	BMI > 30.0: *M* (*SD*)	*ANOVA*: *F* (*p*)
*The Multidimensional Body-Self Relations Questionnaire*					
Appearance Evaluation	7–35	24.28 (6.69)	24.44 (5.50)	19.97 (5.81)	15.37 (.000)
Appearance Orientation	12–60	43.14 (8.27)	43.63 (7.33)	43.34 (8.89)	.11 (.895)
*The Body Esteem Scale*					
Sexual Attractiveness	13–65	46.61 (7.81)	47.31 (7.53)	46.99 (8.66)	.23 (.798)
Weight Concern	10–50	34.93 (8.01)	33.21 (8.20)	27.19 (8.55)	19.21 (.000)
Physical Condition	9–45	37.36 (9.61)	38.06 (9.24)	33.68 (8.24)	5.94 (.003)

In the next stage of our analysis we verified which characteristics of body image (BSE) correlate with overall perception of attractiveness or activities towards improvement of the latter (MBSRQ). The results of inter-correlation analysis are summarized in Table 2. We found that the perception of *Sexual Attractiveness* is the most strongly correlated with *Weight Concern*. It is of note that this correlation was the strongest in the case of women with BMI < 17.5. The correlation was weaker in women with “ideal BMI” and decreased to *r* =. 27 in obese women. Moreover, *Weight Concern* correlated with *Physical Condition* in all groups; however, in contrast to *Sexual Attractiveness*, this correlation was inversed. The strongest relationship was documented in obese women and the weakest in severely underweight participants. Finally, *Weight Concern* correlated with *Appearance Evaluation* in all groups; this association in extremely slim and obese women was stronger than in the case of “ideal BMI” group. The correlation between *Appearance Evaluation* and *Sexual Attractiveness* pertained mostly to underweight women.

**Table 2:** Correlation between the results of BSE and MBSRQ in women with various BMI.

Scale	BMI	MBSRQ		BES		
		Appearance Evaluation	Appearance Orientation	Sexual Attractiveness	Weight Concern	Physical Condition
*The Multidimensional Body-Self Relations Questionnaire*						
	<	**–**				
Appearance Evaluation	n	**–**				
	>	**–**				
	<	.20	**–**			
Appearance Orientation	n	.20 (.010)	**–**			
	>	.34 (.004)	**–**			
*The Body Esteem Scale*						
	<	.43 (.000)	.14	**–**		
Sexual Attractiveness	n	.17 (.030)	.08	**–**		
	>	.10	-.11	**–**		
	<	.44 (.000)	.07	.56 (.000)	**–**	
Weight Concern	n	.27 (.000)	-.08	.39 (.000)	**–**	
	>	.42 (.000)	-.01	.27 (.022)	**–**	
	<	-.07	-.09	.08	.26 (.018)	**–**
Physical Condition	n	.06	-.08	.09	.42 (.000)	**–**
	>	.18	-.07	.13	.54 (.000)	**–**

*Note*: *r* (*p*); < – extremely slim women, n – “ideal BMI”, > – obese women

Our principal hypothesis pertained to the assumption that narcissistic traits are important determinants of self-assessed attractiveness of examined women with various BMI. Therefore, we verified if the power of these relationships differed depending on BMI group. However, first of all we tested if women with dissimilar BMI differ with regards to the intensity of narcissistic traits. The homogeneity of variance of all analyzed variables was confirmed with the *Levene Test* (0.34 < *F* < 1.18,. 310 < *p* <. 712). The results of the analysis of variance (*ANOVA*) suggested that women with diverse BMI differed significantly with regards to *Need for Admiration* and *Vanity* (Table 3). However, the intergroup analysis (*Tukey post-hoc Test*) revealed that women with “ideal” BMI differed from obese women only in terms of *Need for Admiration* (*p* =. 041) and *Vanity* (*p* =. 033).

**Table 3:** Differences in the intensity of narcissistic traits in women with different BMI.

Scale	BMI < 17.5: *M* (*SD*)	21.7 < BMI < 22.7: *M* (*SD*)	BMI > 30.0: *M* (*SD*)	*ANOVA*: *F* (*p*)
*Narcissistic Personality Inventory*				
Need for Admiration	32.66 (8.58)	33.66 (7.30)	30.67 (8.49)	3.61 (.028)
Leadership	30.53 (8.48)	31.96 (7.85)	30.78 (7.56)	1.14 (.322)
Vanity	14.82 (4.17)	15.79 (3.75)	14.19 (3.64)	4.83 (.009)
Self-Sufficiency	23.99 (4.77)	25.23 (3.71)	24.58 (4.73)	2.50 (.083)

The values of correlation between narcissistic traits and physical self-assessment are presented in Table 4. The correlations were the strongest in the case of slim women. All analyzed narcissistic traits correlated positively with *Sexual Attractiveness*, *Weight Concern*, and *Appearance Evaluation*. Furthermore, there was a significant relationship between *Vanity* and *Appearance Orientation*. The latter positive correlation (i.e. *Vanity* vs. *Appearance Orientation*) was documented in all groups of women. The lowest number of statistically significant correlations between the narcissistic traits and self-assessed image pertained to obese women.

**Table 4:** Correlations between the scales of self-image (BSE and MBSRQ) and the narcissistic traits (NPI) of women with various BMI.

Scale	BMI	*NPI*			
		Need for Admiration	Leadership	Vanity	Self-Sufficiency
*The Multidimensional Body-Self Relations Questionnaire*					
	<	.45 (.000)	.50 (.000)	.62 (.000)	.48 (.000)
Appearance Evaluation	n	.25 (.001)	.33 (.000)	.46 (.000)	.32 (.000)
	>	.30 (.013)	.30 (.013)	.54 (.000)	.19
	<	.23 (.031)	.19	.42 (.000)	.23 (.031)
Appearance Orientation	n	.25 (.001)	.17 (.027)	.31 (.000)	.07
	>	.17	-.05	.29 (.015)	-.13
*The Body Esteem Scale*					
	<	.43 (.000)	.41 (.000)	.46 (.000)	.34 (.001)
Sexual Attractiveness	n	.29 (.000)	.24 (.002)	.27 (.000)	.15
	>	.21	.20	.21	.26 (.032)
	<	.37 (.000)	.39 (.000)	.42 (.000)	.27 (.013)
Weight Concern	n	.21 (.007)	.14	.24 (.002)	.12
	>	.11	.11	.38 (.001)	.16
	<	.13	.24 (.028)	.05	.13
Physical Condition	n	.08	.09	-.01	.00
	>	.10	.09	.15	.37 (.002)

*Note*: *r* (*p*); < – extremely slim women, n – “ideal BMI”, > – obese women

**Fig 2:**
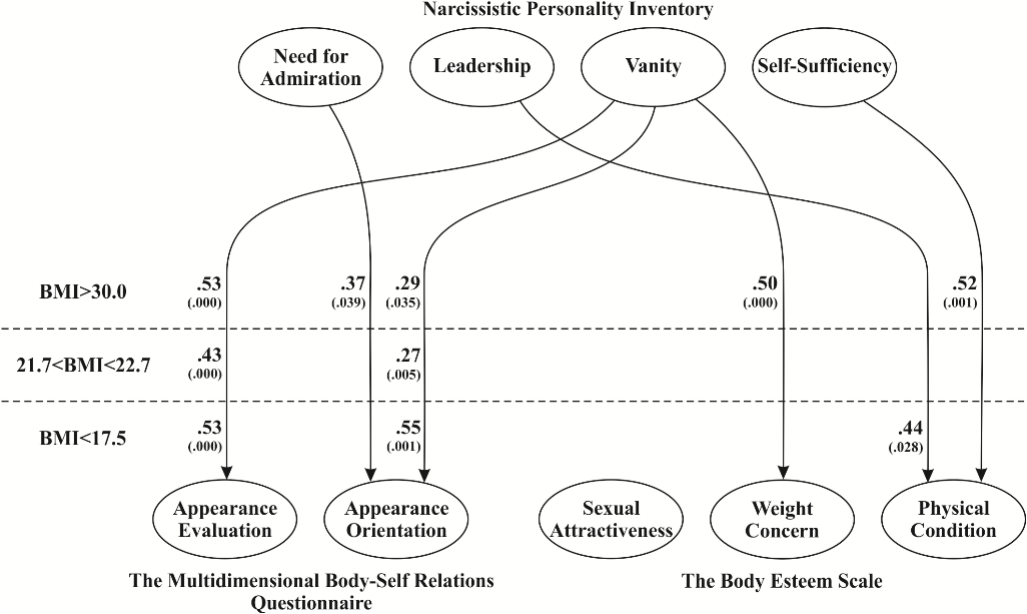
Analysis of multiple regression: influence of narcissistic traits on the self-perceived attractiveness of women from three BMI categories (obesity, “ideal BMI”, slim).

Next we used multiple regression analysis to verify which narcissistic traits exert the strongest effect on the self-assessed attractiveness of women with different BMI. The analyses were performed separately for each self-attractiveness scale and each of BMI categories. The predictors which proved significant on all analyses are presented in Fig 2. Vanity proved the strongest predictor for both *Appearance Orientation* and *Appearance Evaluation*; importantly, this association was not modulated by the group (BMI level) as confirmed with the use of Indicator Variables in Regression. Moreover, *Need for Admiration* proved the significant predictor of *Appearance Evaluation* in obese women, and dummy variable did not reveal difference in relation to slim women and participants with “ideal” BMI.

The predictors (narcissistic traits) did not form a well-fitted regression model with *Sexual Attractiveness*. *Vanity* modulated *Weight Concern* in obese women but not in those with “ideal” BMI (*β =*. 18, *t* = 1.82, *p* =. 071, dummy variable *t* = 2.12, *p* =. 035). Also the analysis of predictors of *Physical Condition* produced interesting findings: *Self-Sufficiency* proved the significant predictor in the case of obese women but not in the case of slim participants (*β = -*.02, *t* = 0.14, *p* =. 891, dummy variable: *t* = 2.19, *p* =. 029) or those with “ideal” BMI (*β = -*.03, *t* = 0.36, *p* =. 720, dummy variable: *t* = 2.59, *p* =. 010). In contrast, *Leadership* was the significant predictor of *Physical Condition* in slim women but not in obese women (*β = -*.25, *t* = 1.47, *p* =. 148, dummy variable: *t* = 2.53, *p* =. 012).

## Discussion

First of all, it should be emphasized that although our study confirmed the assumption on the significant influence of body weight on the self-perception of body attractiveness, the group that stood out was comprised of obese women. Body weight constitutes one of the strongest predicators of self-satisfaction with one’s body image, and individuals with the highest weight usually represent the lowest level of self-satisfaction [39,76]. Unfortunately, this is not a simple relationship and one can talk about the self-perpetuating spiral of dissatisfaction, since the lower is the self-satisfaction with one’s image, the more frequently he/she perceives his/her body as greater than in reality. Thus, individuals who try to lose weight usually overestimate their size [77,78]. Many studies revealed that contemporary culture stigmatizes obese individuals, and this rule applies both to adults and children [78,79]. Long-term exposure to social stigmatization increases the probability that overweight subjects will internalize negative information about themselves, and can be reflected by the feeling of mental discomfort with regards to one’s body image [80,81]. The important role of long-term exposure and stigmatization was confirmed in studies dealing with the self-evaluation of pregnant women in the third trimester of pregnancy, whose body image was generally positive and associated with the positive self-esteem [82]. Undoubtedly, the final weeks of pregnancy are evidently associated with high body weight and unshapely silhouette, but these changes are not reflected by any negative social evaluation of pregnant woman’s image. The awareness of relatively quick possibility of losing excessive kilograms and expecting a child represent factors that protect pregnant woman against decreased self-evaluation related to her image.

More comprehensive analysis of the level of satisfaction pertaining to various components of beauty revealed that studied women did not differ significantly with regards to their Sexual Attractiveness. This finding is consistent with the assumption made by the authors of BES test [70]. The components of beauty that highlight female sexuality, e.g. the shape of lips, or breasts, are not directly related to body weight. Moreover, in the case of dissatisfaction with their image, they can be corrected with various techniques of makeup or even by means of plastic surgeries [83].

The results regarding *Weight Concern* are particularly interesting since documented intergroup differences reflected significantly lower level of satisfaction in obese women. Although this finding is not surprising since obese women usually declare problems with weight control, as proved by frequent unsuccessful attempts to lose weight [21,77], one should emphasize the high level of weight control experienced by underweight women. This finding seems contradictory to the published research on body image among women with anorexia [70,84], who usually declare low levels of satisfaction with their weight control. It should be noted, however, that although our study included women with BMI below 17.5, none of them was diagnosed with anorexia nervosa. The evidence of underweight or even cachexia (the lowest BMI documented in our group was equal to 14.3) is alone insufficient to diagnose anorexia according to DSM-IV-TR criteria [68] as low body weight should be accompanied by such findings as impaired body image, and the fear of gaining weight or being obese. Since individuals with eating disorders perceive their bodies as foreign entities and both the awareness of their body experiences and attitude towards the body are impaired, they are unable to internalize the objective information on low or reduced body weight. Slim women who participated in our study were aware of their body weight and showed high levels of its control. Moreover, these women scored their *Physical Condition* at a similar level as participants with “ideal BMI”, reiterating the recently frequent finding of a relationship between extremely low body weight and care of physical condition both in anorexia patients and in athletes (e.g. rhythmic gymnastics) without the diagnosis of this disorder [85,86]. The relationships between *Physical Condition* score and general satisfaction with body image or weight control could be interpreted as a positive finding, i.e. as an increasing awareness of the role played by physical activity and healthy diet in determining and maintaining slender and healthy silhouette. However, this interpretation is contradicted by the lack of significant association between the involvement in improving one’s image and physical condition or weight control. This is particularly surprising in the case of obese women; on the other hand, it can be noted that the objectively confirmed overweight is not necessarily associated with active involvement in silhouette improvement. As many as 76% of our women who exercise in a fitness club have normal BMI, and only 15% of them represent overweight or obesity [87]. This is mostly associated with two absolutely different trends promoted in mass media: creating fashion for slim and fit silhouette on one hand, and satisfying all culinary caprices and sedentary lifestyle on the other. Such contradictory information, especially in the case of children and adolescents growing up in the television era, is reflected by the creation of an image of slim person with hamburger in one hand and a bottle of Coca-Cola (obviously, Cola light) in the other, and ingesting a chocolate bar in the case of sudden hunger, obviously with no negative consequences to his/her silhouette [88].

Paradoxically, it is more difficult for obese individuals to decide to lose weight than for slimmer individuals as losing several or several dozen kilograms by evidently overweight individual requires very intensive involvement. Also, despite reducing weight such person is still perceived as fat. Consequently, why should he/she make an effort if it will result solely in being a “slimmer fat person”? In the case of obese persons reducing the number of ingested calories, being on a rigorous diet, or pursuing unrealistic weight correction are not enough; also, the modification of life style, along with resultant improvement in physical fitness and health, are necessary. Health cannot be equaled to the loss of excessive kilograms; slimmer does not necessarily correspond to healthier [77,80]. Frequently, the decision upon initiating weight loss does not result from the factual physiological or medical need; dieting becomes a part of life style where a woman should refuse eating since such behavior affirms her high level of internal control and care of her body.

Feedback received from others, and above all the need for positive assessment, constitute the component of narcissism analyzed as a personality trait. Previous studies revealed a positive correlation between self-assessment of one’s body and narcissism [22,23], and therefore we focused on investigating the relationship between the level of various narcissistic traits and the level of self-satisfaction with body image in women with various BMI. Observed in our study lack of significant differences between underweight women and women with normal weight with regards to theirs narcissism intensity was partially confirmed by other authors [89]. Obese women are characterized by rather low level of self-esteem, and therefore this group does not show high levels of narcissism considered as a personality trait. Obesity is a chronic condition; most obese young women were previously obese girls which exerts significant effect on the development of their personality. More frequently, eating disorders characterized by extremely low body weight, are rather associated with narcissistic defense [24]. However, as previously emphasized, despite extremely low weight, the study group of women with BMI < 17.5 did not satisfy any other diagnostic criterion of anorexia. Therefore, they were not characterized by low self-assessment of body, and anxiety- or escape-like attitude to losing weight [85]. Therefore, this group of our participants comprised slim individuals, who were aware of this fact, and moreover, intentionally chose this appearance.

It is not surprising that *Vanity* was the narcissistic personality trait, which modulated self-perception the strongest. The assumption on this relationship is in fact underlying the theoretical construct of narcissism; it is of note, however, that depending on BMI level, this trait influenced various components of physical attractiveness in our women. Irrespective of body weight, *Vanity* modulated the overall self-satisfaction with one’s appearance; however, only in obese women higher level of *Vanity* seemed to attenuate the *Weight Concern* score. Such association was also revealed in a study on the effectiveness of losing weight after bariatric surgery, in which the level of narcissism correlated inversely with the number of lost kilograms [90]. In contrast, in the case of women with severe underweight, we observed significant association between this trait and involvement in activities aimed at image improvement. It is frequently postulated that slim women, both with no features of anorexia and with diagnosis of this disorder, are heavily involved in sport activity [85,87,91]. It is even postulated that it is involvement in physical activity which is stronger associated with positive body image and positive health perceptions rather than objective health status or involvement in other health practices [92]. Interestingly, in the case of obese women, the enhanced activities aimed at body image improvement result mostly from pursuing social acceptance. The need for being liked by others rather than a simple vanity stimulates them to such activities. We postulate that this is not only associated with the need for improving the attractiveness of body image, but also with the fact that pursuing slimness is socially perceived as a sign of self-control and is even required by others [80]. Such interpretation was confirmed by the association between *Self-Sufficiency* and *Physical Condition*, pointing to the awareness of the relationship between self-competence or self-success and self-assessment of one’s body [39,77].

The relationship between *Leadership* and *Physical Condition* in underweight women should be considered interesting. Although our participants did not satisfy the diagnostic criteria of anorexia, they ascribed significant role to the need for self-control and control over others. This finding constitutes another contribution to ongoing discussion on expanding the definition of eating disorders [85].

Our study provided further evidence confirming that both the objective anthropometric parameters and the level of narcissism are associated with an attitude of young women of their bodily sphere. Having in mind, that one’s attitude to his/her body is defined as early as in the childhood [79,93,94], mostly under the influence of other people [27], the guardians should do their best to prevent a decrease in self-esteem of their children, resulting either from underweight [42] or from excessive attention paid to body image [95].

However, the preventive measures should not be oriented solely at improvement of health, prevention of obesity or underweight, but also center around attenuation of the role of body image as a determinant of narcissism dimensions, such as *Need for Admiration* and *Vanity*. High level of narcissism, which was shown to be inversely correlated with subjective well-being [96], combined with lack of self-acceptance to body image, may constitute an important risk factor predisposing to distortion of the latter or promoting development of eating disorders [39,46,52].

## Conclusions

Body weight constitutes the determinant of satisfaction with body image in young women. Also narcissism, considered as a personality trait, proved to constitute a significant modulator. However, the role of narcissism as a modulator of self-satisfaction with one’s body varies depending on BMI level: extremely underweight women and obese individuals constitute groups in which narcissism has the strongest impact on the self-satisfaction with body.
